# Commercial and Instant Coffees Effectively Lower Aβ1-40 and Aβ1-42 in N2a/APPswe Cells

**DOI:** 10.3389/fnut.2022.850523

**Published:** 2022-03-15

**Authors:** Lifang Zhang, Jessica Cao, Haiqiang Yang, Phillip Pham, Umer Khan, Breanna Brown, Yanhong Wang, Tarek Zieneldien, Chuanhai Cao

**Affiliations:** ^1^Department of Neurological Rehabilitation, The Affiliated Brain Hospital of Guangzhou Medical University, Guanzhou, China; ^2^Department of Kinesiology, Wiess School of Natural Sciences, Rice University, Houston, TX, United States; ^3^Department of Pharmaceutical Sciences, Taneja College of Pharmacy, University of South Florida, Tampa, FL, United States; ^4^Department of Neurology, College of Medicine, University of South Florida, Tampa, FL, United States

**Keywords:** coffee, Alzheimer's disease, caffeine, beta amyloid, toxicity

## Abstract

**Background:**

Alzheimer's disease (AD) is a multifactorial neurological disease with neurofibrillary tangles and neuritic plaques as histopathological markers. Due to this, although AD is the leading cause of dementia worldwide, clinical AD dementia cannot be certainly diagnosed until neuropathological post-mortem evaluation. Coffee has been reported to have neurologically protective factors, particularly against AD, but coffee brand and type have not been taken into consideration in previous studies. We examined the discrepancies among popular commercial and instant coffees in limiting the development and progression through Aβ1-40 and Aβ1-42 production, and hypothesized that coffee consumption, regardless of brand or type, is beneficial for stalling the progression and development of Aβ-related AD.

**Methods:**

Coffee samples from four commercial coffee brands and four instant coffees were purchased or prepared following given instructions and filtered for the study. 5, 2.5, and 1.25% concentrations of each coffee were used to treat N2a/APPswe cell lines. MTT assay was used to assess cell viability for coffee concentrations, as well as pure caffeine concentrations. Sandwich ELISA assay was used to determine Aβ concentration for Aβ1-40 and Aβ1-42 peptides of coffee-treated cells.

**Results:**

Caffeine concentrations were significantly varied among all coffees (DC vs. MDC, PC, SB, NIN, MIN *p* < 0.05). There was no correlation between caffeine concentration and cell toxicity among brands and types of coffee, with no toxicity at 0.5 mg/ml caffeine and lower. Most coffees were toxic to N2a/APPswe cells at 5% (*p* < 0.05), but not at 2.5%. Most coffees at a 2.5% concentration reduced Aβ1-40 and Aβ1-42 production, with comparable results between commercial and instant coffees.

**Conclusion:**

All coffees tested have beneficial health effects for AD through lowering Aβ1-40 and Aβ1-42 production, with Dunkin' Donuts® medium roast coffee demonstrating the most consistent and optimal cell survival rates and Aβ concentration. On the other hand, Starbucks® coffee exhibited the highest cell toxicity rates among the tested coffees.

## Introduction

Alzheimer's Disease (AD) is a neurodegenerative disease that is marked by the gradual deterioration of memory and cognition, among other cognitive impairments, which are attributed to extracellular aggregates of Aβ plaques and intracellular neurofibrillary tangles of hyperphosphorylated tau protein (1, 2). AD is the most common form of dementia and is the sixth leading cause of death in the United States (3). Although the mortality rates of other illnesses (heart disease and cancer) have maintained a steady decline over the two past decades, AD-related deaths have increased over 140% in the same timeframe (4). Approximately 6 million Americans are currently living with AD, and this number is expected to rise to nearly 13 million by 2050 (3, 5, 6). These statistics are mirrored by an increasing economic burden; the Alzheimer's Association estimates that in 2018, AD and other forms of dementia will cost the United States $277 billion (7). With the aging population of Baby Boomers, this number is expected to rise to nearly $1.1 trillion by 2050 (6). Furthermore, an estimated 16.1 million Americans provide unpaid assistance for patients with AD and other forms of dementia (3). This unpaid care primarily comes from loved ones of patients, who are consequently financially and emotionally burdened by the disease.

Aging is the most important risk factor for AD (and the most prevalent among those over the age of 65), but the symptoms of AD are not characterized as part of the normal aging process (8). While AD is frequently diagnosed by decreased memory function, many other symptoms such as confusion and behavioral changes are also common features (9). Although there is no distinct set of variables that leads to AD progression, certain factors such as lifestyle and genetics may play a significant role in the onset and severity of the disease (10). Adequate sleep, high-fat diets, regular exercise, and smoking cessation are lifestyle factors that are associated with the prevention of AD progression (11–14). In terms of genetics, the isoform of the apolipoprotein E (ApoE) gene—such as the ApoE4 carrier—is a particularly significant contributor to the development of late onset of AD, with carriers of the ApoE4 gene being five times more likely to develop late-onset AD than those without (15–17). Additionally, individuals with Down syndrome are at greater risk for AD because they have three copies of chromosome 21 (the locus of APP gene), which results in a greater production of beta amyloid (Aβ) compared to normal control (18, 19). However, genetic factors only account for <5% of AD diagnoses, and factors such as lifestyle likely contribute more significantly to disease progression (20–26).

Although there is currently no definitive pathological factor of AD, the abnormal accumulation and aggregation of Aβ and phosphorylated Tau (p-Tau), also known as plaques and tangles, have been observed as a potential cause (27–33). The most widely accepted and recognized explanation for AD development is the Aβ hypothesis, or the Amyloid Cascade hypothesis (34–36). Aβ is a highly hydrophobic and sticky peptide derived from a protein known as amyloid precursor protein (AβPP). The AβPP gene is located on chromosome 21, which may explain the more rapid progression of AD among people with Down syndrome. Aβ accumulates in the brain with aging, and clusters of Aβ form oligomers, which gradually accumulate to form plaques (27, 28). These plaques can disrupt cellular communication and activate immune cells, resulting in neuronal death, neuroinflammation, and oxidative stress which may result in memory impairment (37–40). Neuroimaging through PET/CT scans have correspondingly shown that Aβ deposition levels are directly correlated to the progression of AD (41–45).

The lack of a defining cause for AD is coupled with the absence of a cure. Current drugs work to increase synaptic neurotransmitters, which are not related to the major pathological hypotheses of AD (Aβ or Tau) (46). All FDA-approved AD medications are also only palliative and are not entirely effective for all patients, with limited long-term efficacy and potentially adverse side-effects (47). Given that the average life span of an individual following an AD diagnosis is about 7–10 years, patients are likely to rely on medicine for the long term (48). Therefore, it is crucial that a long term, low-toxicity, and cost-effective method to treat or even prevent AD is discovered. Research has demonstrated that coffee may pose as a potential substance for AD treatment or prevention (49–56).

Coffee is considered a staple among most western countries, and the consumption of coffee has continuously increased in recent years (57, 58). Its complex structure of over 2,000 compounds—most notably caffeine, chlorogenic acids, and diterpenes—enables coffee to have countless benefits, ranging from neuronal protection, to decreased heart disease risk and lower mortality rates (59–64). Caffeine, the most recognized compound in coffee, is known for its stimulating effects on the central nervous system, as well as its beneficial effects on memory (65). Arendash et al. have demonstrated the specific benefits of caffeine in coffee when mice were consistently fed caffeine daily, particularly among older mice exhibiting AD characteristics such as memory loss. Mice were given a memory task of navigating through a water maze before and after daily caffeine consumption. Older mice who consumed caffeine were able to navigate the water maze as effectively as the younger mice, and exhibited reduced quantities of Aβ (55). Since caffeine is a significant molecule in coffee, coffee consumption may yield similar results. Other studies have shown that coffee stalls AD progression and reduces Aβ production with greater efficacy compared to pure caffeine or decaffeinated coffee, indicating that other compounds in coffee function synergistically with caffeine (55, 66, 67). Human studies have also demonstrated that habitual coffee consumption plays a significant role in stalling the progression of cognitive decline (68, 69). Eskelinen et al. reported that participants who consumed 2 or less cups of coffee were reported as the group with the highest occurrence of dementia and AD later in life compared to those who consumed greater quantities (70, 71). Consistent and long-term moderate coffee consumption is also associated with a decreased risk of developing AD and other forms of dementia (72, 73).

Most research reports use caffeine level as the sole major indicator when assessing coffee function, with the assumption that caffeine level is relatively constant across similar types of coffee (i.e., same roast level). However, the species of, and geographic locations in which coffee beans are grown likely results in variations in the coffee that is ultimately consumed. Furthermore, the roast time and preparation methods also influence the levels of active compounds in coffee. Different brands of coffee are processed and brewed in various ways, ultimately resulting in differences in coffee bean quality and the concentrations of different compounds (74–76). Despite these discrepancies, the variability among different coffee brands has never been compared. Although we believe that coffee consumption, regardless of type or brand, is beneficial for stalling the progression and development of Aβ-related AD, the discrepancies among popular commercial and instant coffees in limiting the development and progression through Aβ1-40 and Aβ1-42 production were examined.

## Materials and Methods

### Coffee Preparation

Dunkin' Donuts®, McDonald's®, Panera Bread®, and Starbucks®–referred to in this manuscript as DC, MDC, PC, and SBC—were selected as the four brands of commercial coffee, as they are several of the most popular commercial coffee brands and are widely available across the United States. Folgers Coffee®, Maxwell House®, Nescafe Classico®, and Starbucks® instant coffees— referred to in this manuscript as FIN, MIN, NIN, and SBIN—were also selected based on popularity, availability, and compatibility to match with the commercial coffees (medium roast).

One small cup of medium roast, black coffee was purchased from Dunkin' Donuts®, McDonald's®, Panera Bread®, and Starbucks® (the specific number of ounces varies per fast food chain). Packets of instant coffees were also purchased from local grocery stores and prepared by following the instructions provided on the packaging. Ten milliliter of each coffee was filtered with 0.22 μM syringe filters, collected in a 50 mL tube, and frozen at −80°C for future application. Three different concentrations of each coffee were obtained using a serial dilution with tissue culture medium, yielding final concentrations of 5, 2.5, and 1.25%. The remaining solution was frozen and stored for future use.

### Cell Line Selection

N2a/APPswe cells were acquired through the American Type Culture Collection (ATCC) prior to the start of experimentation. The N2a/APPswe cell line was selected for this study and is derived from inserting the human mutant APP gene into mice neuroblastoma cells (N2a). N2a/APPswe cells are considered the cell model for Aβ-related AD since the cells secrete human Aβ40/42 peptides, which are believed to be a major pathological factor of AD.

### Antibodies and ELISA Kits

Antibodies and ELISA kits were ordered from MegaNano Diagnostics Inc. (Tampa, FL, USA) and used for three trials to ensure consistent results. Aβ1-40, Aβ1-42, and Aβ1-40 vs. 1-42 ratio were used to evaluate treatment benefits and efficacy. Caffeine level was detected by using a competitive ELISA assay manufactured by Neogen (Lansing, MI, USA).

### Treatment and Sample Collection

On the first day, 8,000 cells/100 μL were seeded into each well of a 96-well plate and cultured in the tissue culture incubator at 37°C for 24 h. The next day, concentrations of 5, 2.5, and 1.25% coffee solutions were prepared as 2× solutions, and 100 μL of each 2× solution was added to the designated wells and incubated at 37°C and 5% CO_2_ for 36 h. After treatment, 100 μL of medium was transferred from each well into a new plate and stored at −80°C for future assays (Aβ1-40 and 1-42 ELISA).

### MTT Assay for Cell Toxicity

At the end of cell treatment, samples from each well were removed for Aβ analysis. MTT reagent was pre-warmed at 37°C, and 10 μL was then added to each well and incubated for 4 h. After incubation, 50 μL of stabilizer was added to each well and incubated overnight at 37°C. The plate was read at an absorption wavelength of 580 nm, and the percentage of live cells was calculated in reference to a no-coffee-treated control.

### Sandwich ELISA Assay for Aβ1-40 and 1-42 Detections

The concentrations of Aβ40/42 were measured by the Aβ1-40 and 1-42 specific sandwich ELISA kit (Mega Nano Biotech. FL, USA). Each well of a 96 well plate was coated by 50 μl G1-42 (goat anti-human Aβ 1-42) antibody [AB-001, MegaNanobiotech Inc. (MN Inc.), FL] diluted to 1XPBS 10 μg/ml, and incubated overnight at 4°C. The plate was then washed 5 times and blocked by adding 200 μl blocking buffer at 37°C for 1 h. After washing the plate, 50 μl diluted detection antibodies anti-Aβ40 (Ab40-002, MN Inc., FL) or anti-Aβ42 (AB42-002, MN Inc., FL) were mixed with either 50 μl diluted peptide standard solution or diluted samples in a preparation plate, and added into each well of the assay plate. Plates were then incubated at 4°C for overnight. After washing, 100 μl of diluted secondary antibody was added to each well and incubated for 45 min on an orbital shaker at room temperature. The plate was washed 4 times, and TMB peroxidase substrate (Surmodics Cat: TMBS-1000) was added to each well and incubated at room temperature for 10 min. The reaction was stopped by adding 100 μl/well of 0.4M H2SO4. Absorbance at 450 nm was read with a BioTek Synergy H4 microplate reader. The concentration was calculated upon the peptide standard.

### Competitive ELISA for Caffeine Level Detection

Caffeine concentrations were measured using ELISA Kits from Neogen (WI, USA), following the manufacturer's protocol. The enzyme conjugate solution was prepared by diluting the 180X enzyme conjugate stock in a 1:180 dilution in the EIA buffer provided, and the remainder of the assay was performed as documented in Cao et al. (68).

### Data Analysis and Graphing

All data were graphed using GraphPad Prism 8.0 software and analyzed using two-way ANOVA (simple effects within rows) followed by Tukey's adjusted pairwise comparisons analysis among groups, with alpha set at 0.05.

## Results

Note: Commercial coffees: DC = Dunkin' Donuts®, MDC = McDonald's®, PC = Panera Bread®, SBC = Starbucks® instant coffees: FIN = Folgers®, MIN =M axwell®, NIN = Nestle®, SBIN = Starbucks®.

### There Are Significant Differences in Caffeine Level Across Different Coffee Brands

**Figure 1:**
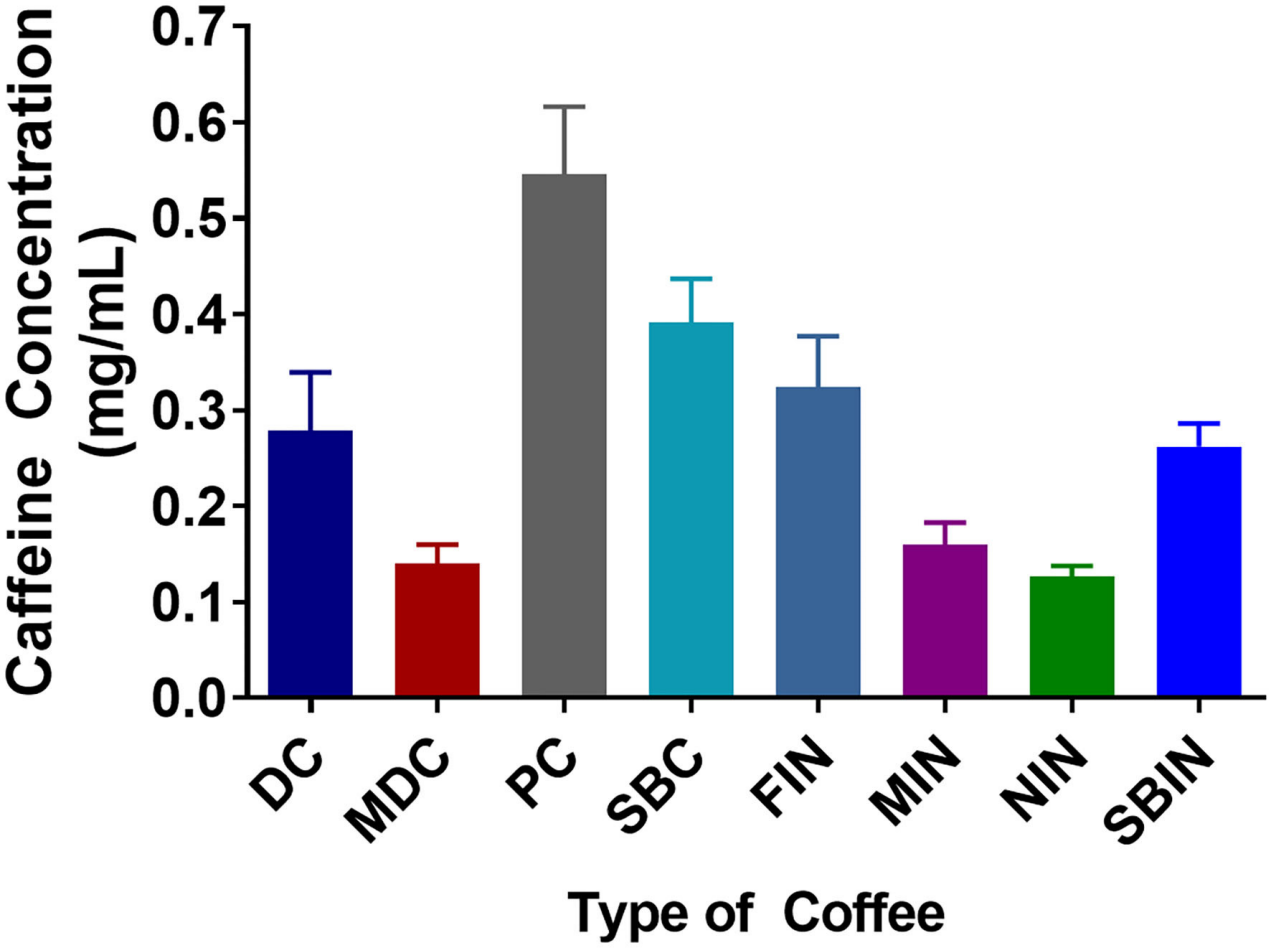
Caffeine concentrations among different commercial coffees and instant coffees were measured using a competitive ELISA assay. Each caffeine concentration was measured in 4 replicates (*N* = 4).

In general, significant differences were noted when examining the different caffeine concentrations among different commercial and instant coffees *via* competitive ELISA (Figure 1). PC and SBC had significantly greater caffeine concentrations compared to DC and MDC, and FIN and SBIN had significantly greater caffeine concentrations compared to MIN and NIN (DC vs. MDC, PC, SB *p* < 0.05; FIN vs. MIN, NIN *p* < 0.05; MIN vs. SBIN *p* < 0.05). Across all coffees, PC, SBC, and FIN contained considerably more caffeine than the other coffees tested (DC vs. MDC, PC, SB, NIN, MIN *p* < 0.05; MDC vs. PC, SBC, FIN *p* < 0.05; PC vs. all *P* < 0.05; SBC vs. all instant coffees except FIN *p* < 0.05; FIN vs. MIN, NIN *p* < 0.05; MIN vs. SBIN *p* < 0.05; MIN vs. SBIN *p* < 0.05). Panera had the highest caffeine level (0.546 mg/ml) among all tested commercial coffees, and McDonald's contained the lowest (0.140 mg/ml). Folgers instant coffee contained the highest caffeine level (0.324 mg/ml) among all tested instant coffees, with Nestle containing the lowest level (0.126 mg/ml).

### Cell Toxicity Is Different for Each Coffee and Is Independent of Caffeine Level

**Figure 2:**
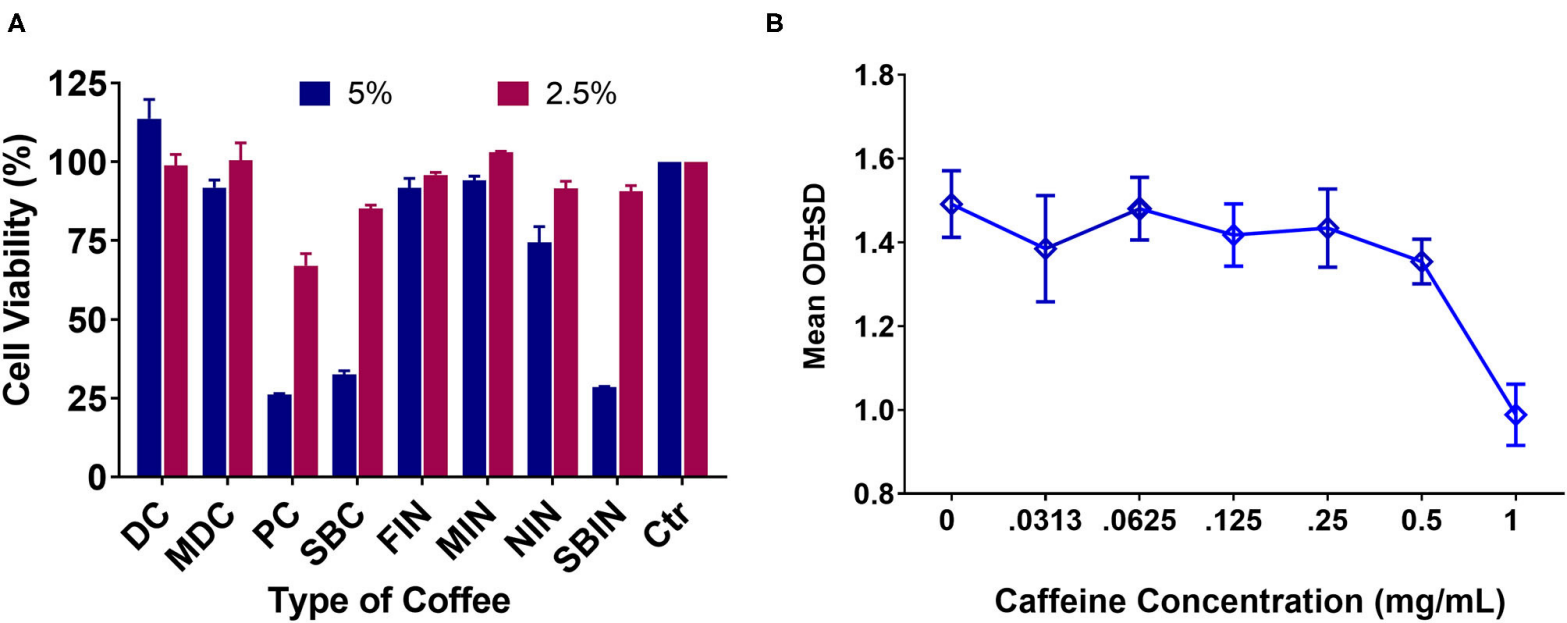
Cell toxicity *via* MTT assay of different coffees and pure caffeine at different concentrations in N2a/APPswe cells (*N* = 4). **(A)** Cell toxicity results of commercial coffees and instant coffees at 5 and 2.5% concentrations. Cell control is set at 100% viability, so any results lower than 100% considered toxic and results higher than 100% showing potential for cell proliferation. **(B)** Pure caffeine toxicity to Na2/APPswe cells *via* MTT assay.

Cell toxicity *via* MTT assay of the different coffees revealed that DC at 5% can significantly increase cell proliferation compared to the no-treatment control group (*p* < 0.01), and MIN showed little to no toxicity to cells at 5% vs. the control group (*p* > 0.05). All other tested coffees are toxic to N2a/APPswe cells at 5% (*p* < 0.05 compared to control). In general, coffee concentration showed much less toxicity at 2.5% or lower. Only PC and SBC, and SBIN are still toxic at 2.5%, while MIN promoted cell proliferation at 2.5% (*P* < 0.05; Figure 2A). Consequently, there is significant toxicity at the 1 mg/ml level to N2a/APPswe cells (*n* = 4 per group), but little to no toxicity at 0.5 mg/ml and lower (Figure 2B).

### Commercial Coffee Can Inhibit Aβ Production in N2a/APPswe Cells

**Figure 3:**
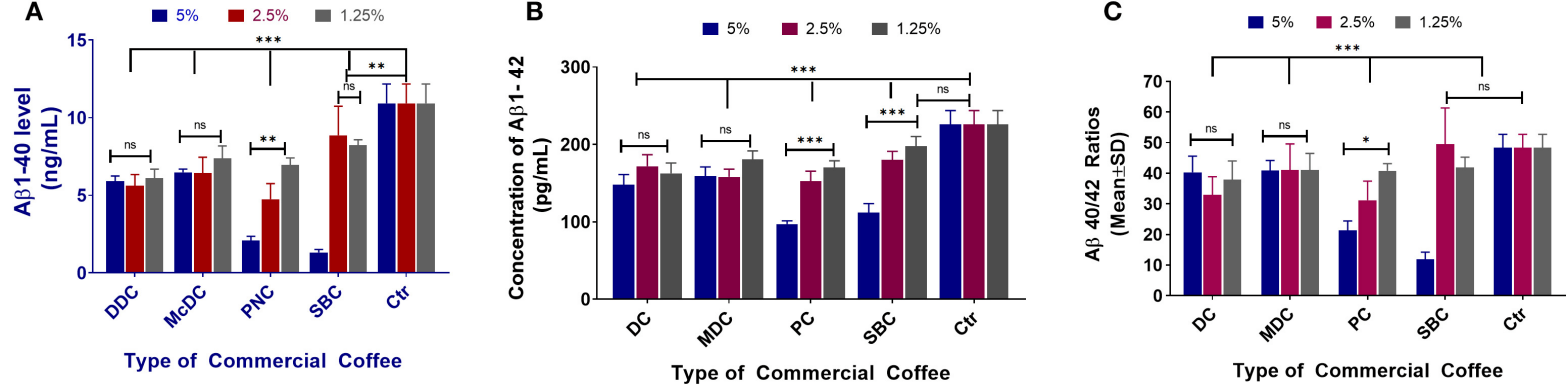
Aβ1-40 **(A)**, Aβ1-42 **(B)**, and Aβ40-42 ratio **(C)** sandwich ELISA assay results of N2a/APPswe cells treated with 4 different commercial coffees (*N* = 4). In the N2a/APPswe cell lines, all commercial coffees had the ability of decreasing Aβ1-40 levels among all concentrations tested (***p < 0.001), without significant differences in concentration (*p* > 0.05) **(A)**. Regarding the Aβ1-42 levels for each commercial coffee, DC decreased Aβ1-42 levels among all tested concentrations (***p < 0.001), with the 5% concentration showing greater effectiveness than the 2.5% (*p* < 0.05) **(B)**. Regarding the Aβ 40/42 ratio changes post-treatment, DC 2.5 and 1.25% concentrations had significantly lower Aβ1-40/1-42 ratios (***p < 0.001 and *p < 0.05) between all 4 coffees, no significant differences were noted between the concentrations (*p* > 0.05) **(C)**.

All commercial coffees showed the potential to decrease Aβ1-40 levels in the N2a/APPswe cell line (Figure 3A). DC and MDC decreased Aβ1-40 levels among all the tested concentrations (^***^p < 0.001), with no significant differences between each concentration (*p* > 0.05). PC lowered Aβ1-40 among all tested concentrations (^***^p < 0.001), with significant differences between each concentration (^***^p < 0.001); SBC lowered Aβ1-40 among all tested concentrations (^**^p < 0.01), with significant differences between 5% compared to 2.5 and 1.25% concentrations (^***^p < 0.001) (Figure 3A). DC decreased Aβ1-42 levels among all tested concentrations (^***^p < 0.001), with the 5% concentration being more effective than the 2.5% (*p* < 0.05); MDC lowered Aβ1-42 among all tested concentrations (^***^p < 0.001), with no significant differences among all concentrations (*p* > 0.05); PC lowered Aβ1-42 among all concentrations (^***^p < 0.001), with the 5% concentration being significantly more effective than the 2.5 and 1.25% concentrations (^***^p < 0.001); SBC lowered Aβ1-42 levels among all tested concentrations (^**^p < 0.01), and the 5% concentration significantly lowered Aβ1-42 levels when compared to the 2.5 and 1.25% concentrations (^***^p < 0.001; Figure 3B). Aβ 40/42 ratio changes post-treatment (Figure 3C). DC 2.5 and 1.25% concentrations had significantly lower Aβ1-40/1-42 ratios (^***^p < 0.001 and ^*^p < 0.05) among all 4 coffees with no significant differences among all concentrations (*p* > 0.05); MDC did not significantly lower the Aβ40-42 ratio among all tested concentrations (*p* > 0.05), with no significant differences between concentrations (*p* > 0.05); PC 5 and 2.5% concentrations significantly lowered the Aβ40-42 ratio (^***^p < 0.001), and the 5% concentration significantly decreased the Aβ40-42 ratio when compared to the 1.25% concentration (^***^p < 0.001); SBC 5% concentration significantly lowered the Aβ1-40/1-42 ratio when compared to the control group (^***^p < 0.001), and when compared with SBC 2.5 and 1.25% (^***^p < 0.001).

### Instant Coffee Can Also Lower Aβ Production in N2a/APPswe Cells

**Figure 4:**
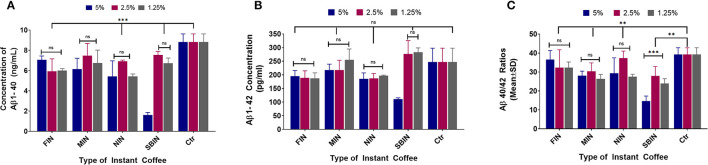
Aβ1-40 **(A)**, Aβ1-42 **(B)**, and Aβ40-42 ratio **(C)** sandwich ELISA assay results of N2a/APPswe cells treated with 4 different instant coffees, which were purchased and were prepared according to the package instructions (the same method was used to treat N2a/APPswe cells as the commercial coffees) (*N* = 4). In the Aβ1-40 results of each coffee treatment, FIN and MIN decreased Aβ1-40 levels among all tested concentrations (***p < 0.001 and **p < 0.01, respectively), without significant differences among concentrations (*p* > 0.05) **(A)**. The Aβ1-42 levels post-treatment with different instant coffees elucidated that FIN decreased Aβ1-42 among all tested concentrations (*p < 0.05), MIN did not significantly decrease Aβ1-42 levels among the tested concentrations (*p* > 0.05), and NIN at 5 and 2.5% concentrations significantly decreased Aβ1-42 levels (*p < 0.05) **(B)**. Furthermore, FIN did not decrease Aβ40/42 among all tested concentrations (*p* > 0.05); MIN decreased Aβ40/42 among all tested concentrations (*p < 0.05); and no significant differences were noted between the concentrations tested for FIN or MIN (*p* > 0.05); NIN 5 and 1.25% concentrations significantly decreased Aβ40/42 (**p < 0.01), and the 2.5% concentration significantly decreased Aβ40/42 in comparison to the 1.25% concentration (*p < 0.05); SBIN decreased Aβ40/42 among all tested concentrations (**p < 0.01), and the 5% concentration significantly decreased Aβ40/42 compared to the 2.5% (***p < 0.001) and 1.25% (*p < 0.05) concentrations **(C)**.

Figure 4A demonstrates the Aβ1-40 results of each coffee treatment. FIN and MIN lowered Aβ1-40 levels among all tested concentrations (^***^p < 0.001 and ^**^p < 0.01, respectively), with no significant differences among concentrations (*p* > 0.05); NIN lowered Aβ1-40 among all tested concentrations (^***^p < 0.001), and the 2.5% concentration significantly lowered Aβ1-40 compared to the 1.25% concentration (^*^p < 0.05); SBIN lowered Aβ1-40 among all tested concentrations (^**^p < 0.01), and the 5% concentration significantly lowered Aβ1-40 compared to the 2.5 and 1.25% concentrations (^***^p < 0.001). Figure 4B shows the Aβ1-42 levels post-treatment with different instant coffees. FIN lowered Aβ1-42 among all tested concentrations (^*^p < 0.05); MIN did not significantly lower Aβ1-42 levels among all tested concentrations (*p* > 0.05); NIN at 5 and 2.5% concentrations significantly lowered Aβ1-42 levels (^*^p < 0.05); there were no significant differences among all concentrations for FIN, MIN, and NIN (*p* > 0.05); SBIN 5 and 1.25% concentrations lowered Aβ1-42 significantly (^***^p < 0.001 and ^*^p < 0.05), and the 5% concentration significantly lowered Aβ1-42 compared to 2.5 and 1.25% (^***^p < 0.001). Figure 4C demonstrates the ratio of Aβ40/42 post-treatment with different coffees. FIN did not lower Aβ40/42 among all tested concentrations (*p* > 0.05); MIN lowered Aβ40/42 among all tested concentrations (^*^p < 0.05); there were no significant differences among all concentrations for FIN or MIN (*p* > 0.05); NIN 5 and 1.25% concentrations significantly lowered Aβ40/42 (^**^p < 0.01), and the 2.5% concentration significantly lowered Aβ40/42 compared to the 1.25% concentration (^*^p < 0.05); SBIN lowered Aβ40/42 among all tested concentrations (^**^p < 0.01), and the 5% concentration significantly lowered Aβ40/42 compared to the 2.5% (^***^p < 0.001) and 1.25% (^*^p < 0.05) concentrations.

### The Comparison Between Commercial Coffee and Instant Coffee on Aβ Modulation

Note: DD = Dunkin' Donuts®, McD = McDonald's®, PN = Panera Bread®, SB = Starbucks®.

**Figure 5:**
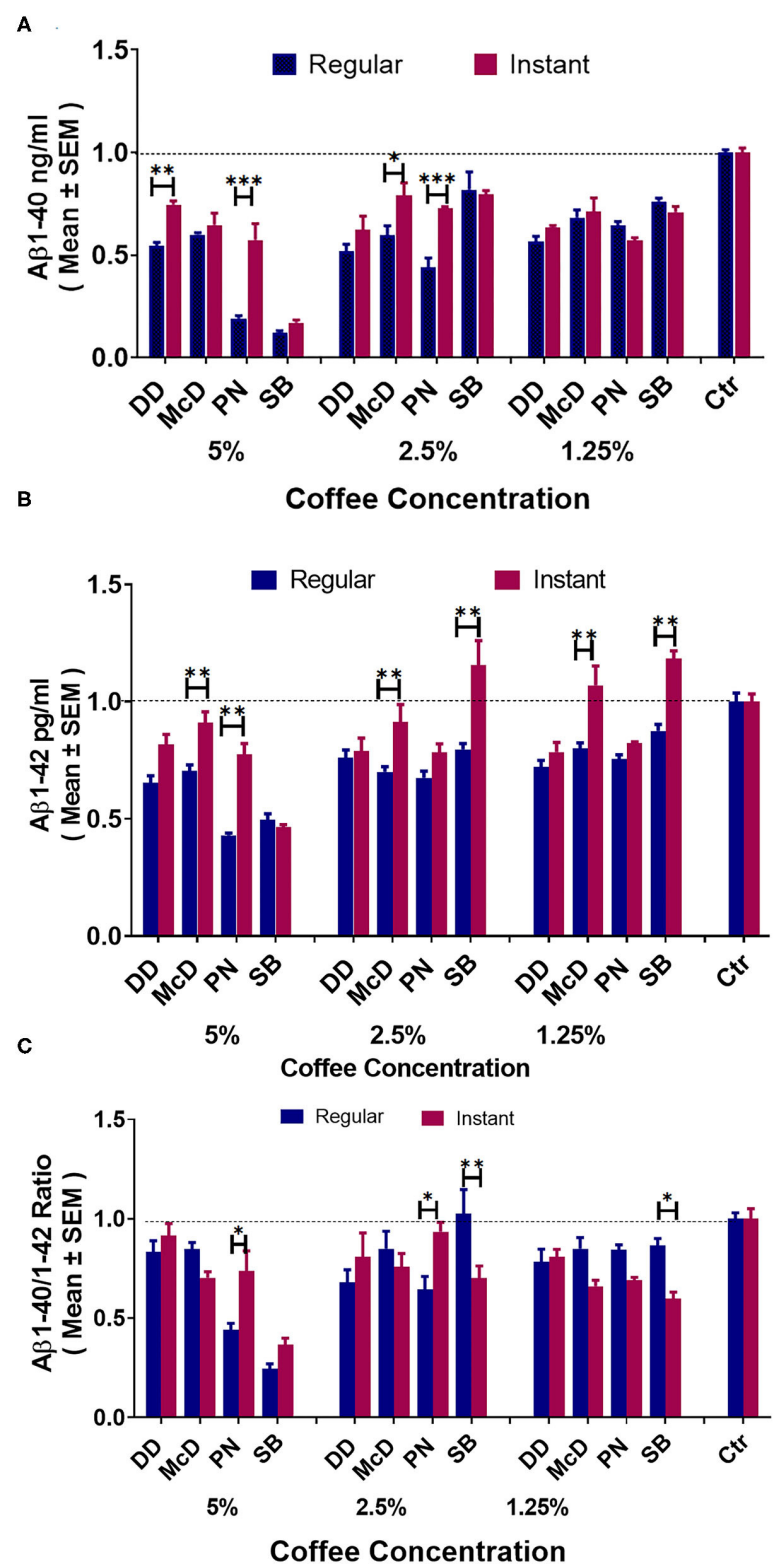
ELISA results of Aβ1-40 **(A)**, Aβ1-42 **(B)**, and Aβ40-42 ratio **(C)** levels in N2a/APPswe cells treated with different commercial coffees vs. their respective instant coffees at different concentrations (*N* = 4). *P < 0.05, **P < 0.01, and ***P < 0.001.

Figure 5A elucidates that all coffee brands show significantly reduced Aβ1-40 production compared to control at all concentrations. At a 5% concentration, DD and PN regular coffee show reduced Aβ1-40 production compared to their brand's instant coffee. At 2.5%, McD and PN regular coffees show significantly reduced Aβ1-40 production compared to the regular coffee. At 1.25%, there were no differences in Aβ1-40 levels across all brands. Figure 5B demonstrates that at 5% concentration, McD and PN regular coffees show significantly reduced Aβ1-42 compared to their brand's instant coffee. At 2.5%, McD and SB regular coffees showed significantly reduced Aβ1-42 compared to their brand's instant coffee. However, SB instant coffee Aβ1-42 is significantly higher than the control group. At 1.25%, McD and SB also showed significantly reduced Aβ1-42 compared to their brand's instant coffee. However, SB instant coffee was significantly higher than control. Figure 5C. At the 5% concentration, PN regular coffee has a significantly lower Aβ40-42 ratio when compared to the brand's instant coffee. Moreover, DD both regular and instant coffee have no significant difference, McD regular coffee compared to control also has no significant difference. At 2.5%, SB regular coffee has a significantly increased Aβ40-42 ratio when compared to the instant coffee. Moreover, McD regular coffee has no significant difference compared to control. Likewise, PN instant coffee also has a significantly increased Aβ40-42 ratio when compared to the instant coffee, but PN instant coffee and SB regular coffee compared to control have no significant differences. At 1.25% McD regular, PN regular, and SB regular vs. control have no significant differences.

## Discussion

Coffee has been one of the most popularly consumed beverage for centuries, yet research regarding its health benefits has only been prevalent in the past few decades (54, 66, 68, 77). An increasing number of publications favor the medical functions of coffee, but the preparation method and correlation between dose and health benefits is still uncertain (74, 76). Furthermore, most research and publications have used self-brewed coffee for experimentation, and the exact brand and roast level have rarely been investigated and reported (66). Thus, little is known about the potential differences in coffee brand and brew type, specifically health consequences relating to AD. Most results on coffee research have also been expanded from caffeine studies, with caffeine level translating to coffee consumption, or with caffeine level being used as a metric for coffee consumption quantity (55). Realistically, it is unlikely that the average coffee consumer would consume the exact amount of suggested caffeine from coffee, so it is critical to know whether caffeine or coffee level is associated with the benefits of AD in coffee drinkers. Additionally, many factors can determine the function of specific coffees, such as production location, manufacture procedures (roast time and temperature), and brewing methods. There is ultimately limited data comparing different brands of coffee, so this study was conducted using the most popular commercial and instant coffees. To do this, different commercial and instant coffees were purchased, and their cell toxicity and anti-Aβ activity was measured in N2a/APPswe cell lines.

While different brands of coffee are similar in terms of convenience and are therefore relatively homogenous, our results demonstrate that there are distinct physiological effects for each coffee, so it cannot be assumed that all brands would have identical effects. Figure 1 indicates that different brands of coffee contain different levels of caffeine. Although caffeine concentrations have been used as a major indicator for coffee despite the thousands of other molecules it contains, overall function is not solely attributed to caffeine content. Other molecules in coffee likely play a pivotal role regarding benefits toward AD, because caffeine level is not correlated to coffee function, as the toxicity assay with coffee revealed a lower cell toxicity than that of pure caffeine. It is also important to note that there is no correlation between caffeine level and cell toxicity, apart from a higher toxicity at a caffeine concentration of 1 mg/ml, while all coffee samples used had caffeine concentrations around or below 0.5 mg/ml (Figure 2B). Although PC and SBC showed higher caffeine levels but lower cell survival rates, DC caffeine concentration was higher than MDC with higher cell survival rates.

Toxicity was instead correlated with coffee level, as only MDC and DC had little to no toxicity to cells at a 5.0% coffee concentration, whereas PC and SBC still exhibited toxicity at 2.5%, albeit to a lesser extent for SBC. There was no cell toxicity at a 2.5% coffee concentration for most commercial and instant coffees (Figure 2A). Coffee consumption should be limited to <5% of the total body fluid level, since a 5% coffee concentration in cell culture was observed to be toxic. Another interesting discovery in our study is that some compounds in coffee may be able to reverse caffeine induced toxicity, because cell toxicity levels for pure caffeine were higher than cell toxicity for coffee containing similar amounts of caffeine.

We have demonstrated that lower and more moderate caffeine concentrations resulted in significantly lowered Aβ concentrations in N2a/APPswe cell lines (Figure 3). While all tested coffees generally lowered Aβ production in N2a/APPswe cells, Starbucks® coffee was the only brand with high toxicity even at a 5% concentration, for both regularly brewed and instant coffee. There is little information on the similarities in origin of all commercial coffee brands, but it can be assumed that many of them originate from similar locations. If this is the case, the procedures used by each corporation to process coffee may serve as a large contributing factor for each brand's impact on neurological health.

The same coffee concentrations of different brands have different functions in cell toxicity and Aβ production, suggesting that further experimentation should be conducted using higher coffee concentrations, specifically for Dunkin' Donuts® coffee to obtain information regarding exact coffee consumption quantity. Prior to beginning formal experimentation, higher concentrations were used for all coffee brands, but Aβ concentrations were not analyzed due to nearly 100% cell death when using Starbucks® and Panera Bread® coffee. As a result of nearly 100% cell death for two of the four commercial coffees being tested, the coffee concentrations were lowered to 5, 2.5, and 1.25% in the following assays.

Another interesting finding from this research is that instant coffee has benefits to AD (lowering Aβ1-40 and Aβ1-42 levels) that are comparable to conventionally brewed commercial coffee (Figure 4). This finding is particularly useful since instant coffee is much easier to prepare and more consistent in quality compared to commercially brewed coffee. Overall, all instant coffees tested lowered Aβ to the non-toxic concentration range, mirroring the commercial coffee results. These findings support previous observations and hypotheses that coffee consumption (regardless of brand or type) is beneficial for stalling the progression and development of AD (68).

In the past, caffeinated coffee has been shown to synergize with another coffee component to elevate plasma GCSF. Long-term GCSF treatment has been shown to enhance cognitive performance in mice models of AD through synaptogenesis, neurogenesis, and recruitment of microglia from the bone marrow (66). Similarly, caffeine, inhibits β- and γ-secretase, which are required for Aβ production (55). Nonetheless, coffee contains an abundance of other components that confer anti-inflammatory and antioxidant activity. As such, the caffeine and non-caffeine components of coffee appear to exert various anti-AD actions.

Dunkin' Donuts® medium roast coffee enabled more consistent and optimal cell survival rates and Aβ concentration compared to the other brands of commercial coffee. Starbucks® coffee generally had the highest cell toxicity rates, suggesting an importance of coffee bean processing as a contributor to health benefit. Overall, all sources of coffee used were shown to have beneficial neurological effects, but the specific function and effectiveness among brands must still be determined. It is worth noting that although coffee was shown to stall AD progression through inhibiting Aβ formation, the amount of coffee consumed in a single serving for this purpose was not determined. All coffees purchased and brewed for experimentation were ~250 ml or 1 cup, so it can be concluded that one-time consumption of this quantity is appropriate.

## Conclusions

For the most part, patients who are diagnosed with Alzheimer's are bound by the disease for the remainder of their lives. Fortunately, coffee generally has no adverse health effects when consumed in normal quantities (2–5 cups per day), which may prove much more beneficial when compared to current pharmaceutical Alzheimer's treatments. Many Americans use commercial (particularly brewed) coffee for convenience purposes, and certain brands are regarded with higher expectations due to the taste of the coffee or the corporation's reputation. We are interested in the role of coffee in Aβ related AD using N2a/APPswe cells to provide scientific guidance for coffee consumers other than caffeine level because millions of Americans consume coffee as an essential factor in their lives. With more knowledge of the distinctions among commercial brands in terms of neurological health benefits, consumers could use our results to make more intuitive decisions to benefit their health.

These findings support previous observations and hypotheses that coffee consumption may act as a long-term preventative measure toward the development of AD. Coffee brand and consumption level are important factors for health benefits, and such benefits are not closely correlated to the caffeine level in the coffee. Instant coffee has similar function to brewed coffee, so it is possible to evaluate dose-related community research using instant coffee. Nonetheless, it is crucial to determine which specific ingredients are synergizing with caffeine to confer such benefits. As such, future studies should focus on the mechanism of coffee in gene regulation, and a long-term cohort human study should be conducted using a quantifiable assay to compare different coffees to guide consumers to reach the maximum health benefits.

## Data Availability Statement

The original contributions presented in the study are included in the article/supplementary material, further inquiries can be directed to the corresponding author.

## Ethics Statement

The animal study was reviewed and approved by the Institutional Animal Care and Use Committee (IACUC) at the University of South Florida.

## Author Contributions

CC and LZ designed research and provided essential materials. JC, HY, PP, UK, BB, and YW conducted research. HY and CC analyzed data. LZ and JC wrote paper. JC and TZ had primary responsibility for editing and revising. All authors have read and approved the final manuscript.

## Funding

This project has been supported by the USF Research Foundation and Florida High Tech Corridor Matching Funds.

## Conflict of Interest

The authors declare that the research was conducted in the absence of any commercial or financial relationships that could be construed as a potential conflict of interest.

## Publisher's Note

All claims expressed in this article are solely those of the authors and do not necessarily represent those of their affiliated organizations, or those of the publisher, the editors and the reviewers. Any product that may be evaluated in this article, or claim that may be made by its manufacturer, is not guaranteed or endorsed by the publisher.
